# Habitat heterogeneity and seasonal dynamics structure mosquito community composition and vector niche differentiation in an intra-urban landscape of southeastern Gabon

**DOI:** 10.1186/s13071-026-07551-6

**Published:** 2026-07-15

**Authors:** Neil Michel Longo Pendy, Arnauld Ondo-Oyono, Boris Kevin Makanga, Miora Felaniaina Andrianandrasana, Ridge Oboulou, Elsa Eyang Assengone, Franck Mounioko, Lynda Chancelya Nkoghe Nkoghe, Ousmane Akone Ella, Marc-Flaubert Ngangue Salamba, Patrick Yangari, Diego Ayala, Patrice Makouloutou Nzassi, Larson Boundenga

**Affiliations:** 1 Centre Interdisciplinaire de Recherches Médicales de Franceville, Franceville, Gabon; 2 Institut de Recherche en Ecologie Tropicale, Libreville, Gabon; 3 Institut Pasteur de Madagascar, Antananarivo, Madagascar; 4 Université des Sciences et Techniques de Masuku, Franceville, Gabon; 5 Institut de Recherche pour le Développement, Montpellier, France

**Keywords:** Mosquito community ecology, Intra-urban heterogeneity, Seasonal dynamics, Environmental gradients, Vector niche differentiation, Urban tropical Africa

## Abstract

**Background:**

Mosquito-borne diseases remain a major public health concern in sub-Saharan Africa, yet the ecological drivers of mosquito community structure in intra-urban environments are poorly understood. This study aimed to assess how habitat heterogeneity, seasonality, and environmental gradients shape mosquito communities in Franceville.

**Methods:**

A longitudinal survey was conducted from July 2023 to July 2024 using 10 CDC light traps deployed weekly for three consecutive nights. Mosquitoes were identified morphologically and environmental, climatic, and urban variables were recorded. Community structure was analysed using diversity indices, principal component analysis (PCA), canonical correspondence analysis (CCA), and Variation Partitioning (VarPar). Species sensitivity to environmental gradients was quantified, and variation partitioning was used to assess the relative contributions of predictor groups.

**Results:**

A total of 6794 mosquitoes representing 24 species across 7 genera were collected. Community composition varied significantly across habitat and season, with higher diversity in forest-associated habitats and more even assemblages in savannah environments. Multivariate analyses revealed distinct ecological responses among genera: *Anopheles* species showed high sensitivity to environmental gradients, whereas dominant *Culex* species exhibited broad ecological tolerance. *Aedes aegypti* was associated with savannah habitats and the rainy periods, while *Ae. albopictus* displayed marked ecological plasticity. Variation partitioning indicated that environmental variables explained a larger share of community variation than climatic and urban predictors, with additional effects from their interactions.

**Conclusions:**

Mosquito communities in southeastern Gabon are primarily structured by fine-scale habit heterogeneity and seasonal dynamics. These findings highlight the importance of integrating ecological complexity and species-specific responses into vector surveillance and control strategies in rapidly urbanising African settings.

**Graphical Abstract:**

**Figure:**
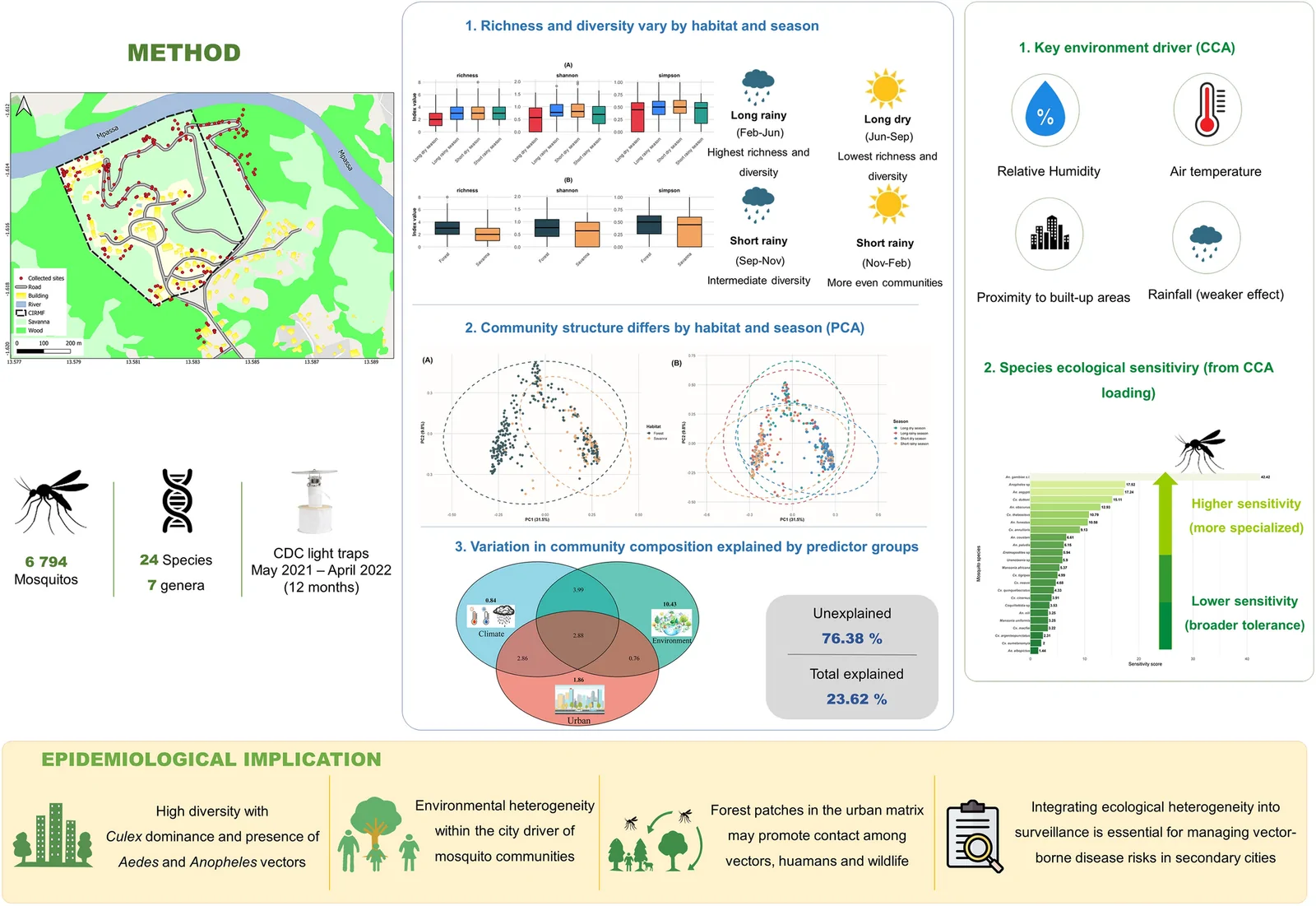

## Background

Mosquitoes are the primary vectors of numerous parasitic [1, 2] and viral diseases [3–6] affecting human populations worldwide, particularly in sub-Saharan Africa [7]. The most epidemiologically important genera are *Anopheles*, *Aedes*, and *Culex*. *Anopheles* mosquitoes primarily transmit human malaria [2], *Aedes* mosquitoes transmit for several major arboviral diseases such as dengue, Zika, chikungunya, and yellow fever [4, 6], and *Culex* mosquitoes mainly transmit lymphatic filariasis and various arboviruses [8]. The spatial distribution, abundance, and ecological preferences of these mosquito taxa critically influence transmission dynamics and outbreak risk. Therefore, understanding their ecological organisation is essential for predicting the emergence or re-emergence of vector-borne diseases and for designing effective, context-specific vector control strategies [9, 10].

Rapid environmental changes in sub-Saharan Africa, such as deforestation [11], agricultural expansion, and accelerating urbanisation, are profoundly altering the habitats of disease vector [12]. These changes create a novel interface among humans, vectors, and pathogens, which often leads to shifts in species dominance, seasonality, and biting behaviour [13]. Mosquito vectors exhibit heterogeneous responses to environmental conditions and habitat heterogeneity: some species remain sensitive to climatic and habitat conditions, while others display considerable ecological plasticity, enabling them to colonise diverse environments [14–16]. These contrasting responses generate marked spatial heterogeneity in mosquito assemblages and, consequently, in local transmission risks.

In Gabon, culicid diversity has been relatively well documented, and the main vector species have been identified [17]. However, most studies have focused on the presence and medical significance of a limited number of key vectors. Considerably fewer have examined how entire mosquito communities are structured along environmental heterogeneity, land use patterns, or climatic variability [18, 19]. This knowledge gap is particularly evident in Franceville, a rapidly growing secondary city in southeastern Gabon. Within its urban landscape, residential areas, institutional compounds, savannah, and remnant forest patches coexist, forming a heterogeneous intra-urban forest-savannah mosaic that is subject to increasing anthropogenic pressure [20]. In such heterogeneous urban landscapes, even subtle environmental variations can substantially influence the composition of mosquito communities and the relative abundance of vector species, thereby directly affecting disease epidemiology.

In this context, a longitudinal eco-entomological survey was conducted to characterise the mosquito community across contrasting habitats within the city of Franceville. Sampling sites included forest patches, savannah environments, and urban areas, to capture fine-scale ecological variability within an intra-urban context. The study had three objectives: (i) to describe seasonal and spatial variation in species composition; (ii) to identify dominant taxa and their abundance patterns; and (iii) to assess the influence of key environmental, climatic, and anthropogenic variables using a multivariate analytical framework combining PCA, CCA, and Variation Partitioning (VarPar).

It was hypothesised that mosquito communities would differ across urban habitats, particularly between forested and savannah environments, due to local environmental heterogeneity and habitat-specific ecological conditions. Furthermore, it was expected that vector species, such as *Anopheles gambiae s.l.*, *Aedes albopictus*, and *Culex quinquefasciatus* would exhibit distinct sensitivities and occupy separate niches within the urban area. Identifying these patterns could provide insights for entomological surveillance and disease prevention in heterogeneous urban landscapes of Gabon.

## Methods

### Mosquito sampling and processing

**Fig. 1:**
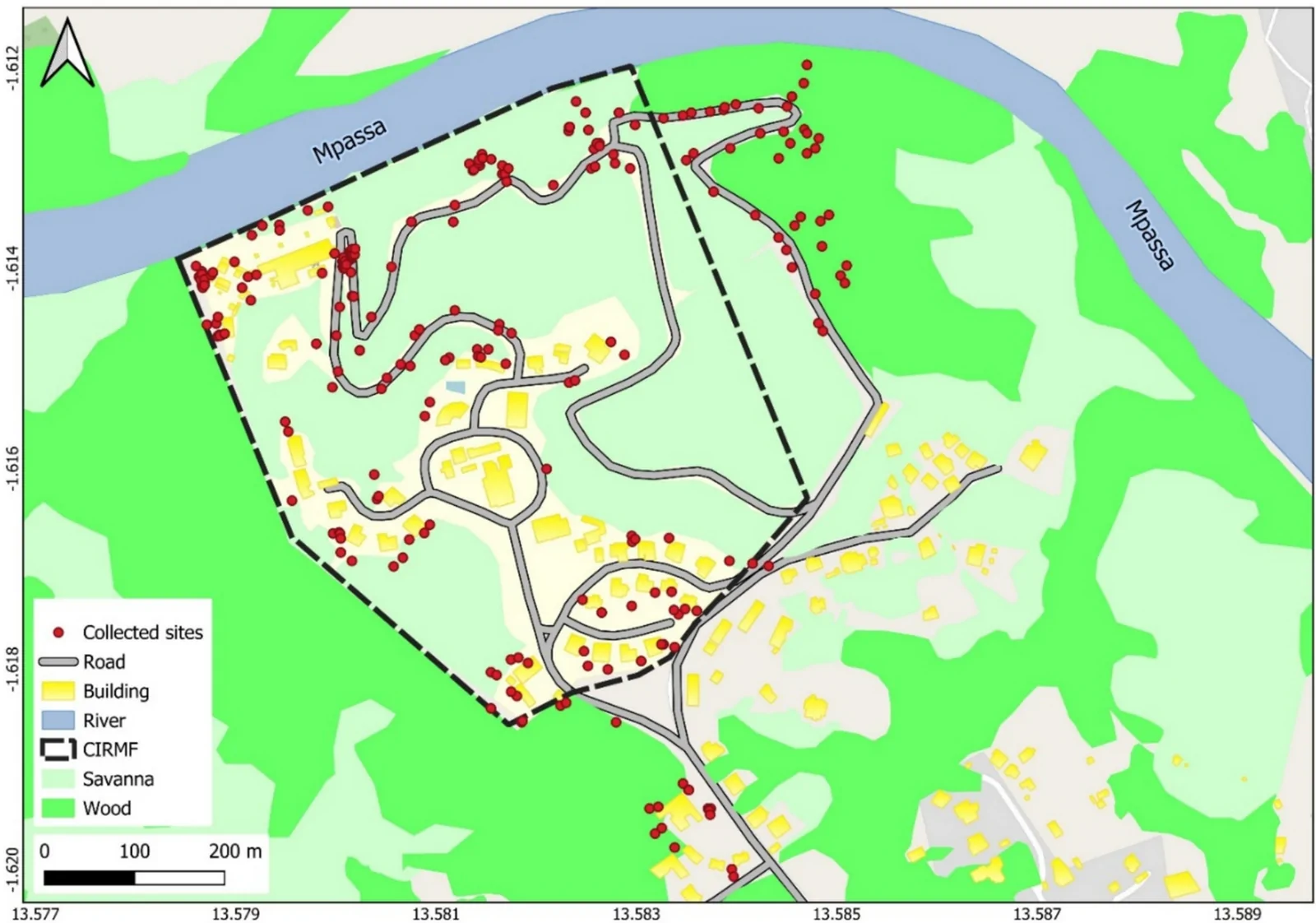
Mosquito collection sites in and around the CIRMF, Franceville

The study design was intended to compare habitat types co-occurring within a heterogeneous intra-urban landscape rather than to represent a formal urban–rural transect. A longitudinal entomological survey was conducted in the city of Franceville from July 2023 to July 2024. Adult mosquitoes were collected using Centers for Disease Control (CDC) Miniature Light Traps (Model 512). Ten traps were deployed weekly across 251 sampling sites at 16:00 and retrieved at 08:00 the following morning for three consecutive nights (Fig. 1). Sites were distributed across contrasting intra-urban habitats including forest patches, savannah environments, and anthropised areas to capture fine-scale ecological heterogeneity within the CIRMF perimeter and adjacent zones. Following collection, mosquitoes were sorted and morphologically identified to species level using standard taxonomic keys for *Culicidae* family in Central Africa and Gabon [21–23]. Identified specimens were then individually preserved in Eppendorf tubes containing silica gel for subsequent analysis.

### Climatic and environmental data

For each location of a CDC Miniature Light Trap and sampling date, a set of environmental and climatic variables was extracted. A circular of 2 km circular buffer was defined around each trapping point to characterise the surrounding landscape and capture local-scale environmental variability that could potentially influence mosquito distribution.

To verify the spatial independence of the sampling units, Moran’s I tests were performed on key environmental and climatic variables (NDVI, temperature, rainfall, relative humidity and distance to buildings) using the *spdep* package in R [24].

Environmental variables included the Normalized Difference Vegetation Index (NDVI), built-up density, the distance to the nearest building and permanent water body, and elevation. NDVI was derived from *MODIS* products (MOD13Q1, with a resolution of 250 m resolution and a 16-day composite) obtained from NASA’s Earthdata platform (https://www.earthdata.nasa.gov/) to characterise vegetation cover. Elevation data were extracted from the Shuttle Radar Topography Mission (SRTM, with an approximate resolution of 30 m resolution) via the United States Geological Survey’s (USGS) EarthExplorer portal (https://earthexplorer.usgs.gov/) to account for topographic variation.

Built-up density was calculated using *OpenStreetMap* data extracted via *QuickOSM* in QGIS (https://www.openstreetmap.org/#map=12/0.4677/9.4280). Building footprints were converted to centroids, integrated into a regular grid and counted per cell to estimate the degree of urbanisation surrounding each sampling site.

Climatic variables including rainfall, air temperature, relative humidity, and soil moisture were obtained from the *ERA5-Land* reanalysis dataset provided by the Copernicus Climate Data Store (https://cds.climate.copernicus.eu/). Climatic variables were extracted from the ERA5-Land reanalysis dataset for each CDC light trap location, based on the sampling sites’ geographic coordinates. ERA5-Land provides climate data with an hourly temporal resolution, enabling short-term environmental variability during mosquito sampling periods to be captured more accurately than with daily averages. These climatic observations were then matched to each mosquito sampling event according to its corresponding trapping location and date. To facilitate the interpretation of temporal variation in mosquito communities, climatic conditions were also summarised according to the seasonal cycle of southeastern Gabon. The regional climate is characterised by four distinct seasons: a long rainy season (mid-February to mid-June), a long dry season (mid-June to mid-September), a short rainy season (mid-September to mid-November), and a short dry season (mid-November to mid-February). Rainfall data extracted from ERA5-Land were aggregated by season to describe the seasonal climate during the study period. As expected, the highest levels of precipitation were observed during the long rainy season (mean = 8.60 mm, median = 8.68 mm, IQR = 6.43), and the lowest levels were observed during the long dry season (mean = 1.67, median = 0.80 mm, IQR = 1.60). Intermediate rainfall levels were observed during the short rainy season (mean = 4.16 mm, median = 1.90 mm, IQR = 3.39) and the short dry season (mean = 6.86 mm, median = 1.91, IQR = 5.15). The relatively high mean rainfall recorded during the short dry season was due to a small number of intense rainfall events, as indicated by the large standard deviation (17.80 mm) and the significant difference between the mean and median values. These predefined seasonal categories were then used as the main temporal framework for subsequent analyses of mosquito diversity, community composition, and species–environment relationships.

### Data processing and statistical analyses

All biological and environmental data were compiled in Microsoft Excel and analysed using R software, version 4.4.3 (R Core Team, 2024). A mosquito domesticity index (DI) was calculated to quantify the affinity of each species of mosquito for human-associated environments. The DI is defined as the proportion of individuals of a given species collected in domestic or peridomestic habitats, divided by the total number of individuals of that species collected across all habitats:

$${\mathrm{DI}}\,\, = \,\,\left( {\text{number of individuals collected in domestic or peridomestic habitats}} \right)/\left( {\text{total number of individuals collected}} \right)$$

Values approaching 1 indicate a strong association with human-modified environments, while values close to 0 reflect a predominance in natural or sylvatic habitats.

Mosquito community diversity was assessed using species richness and two diversity indices: Shannon’s diversity index (H′) and Simpson’s index (D). These were computed using the *diversity* function from the vegan package [25]. These indices provide complementary information on community structure: species richness reflects the number of taxa present; *Shannon’s* diversity index integrates both richness and evenness; and *Simpson*’s index is more sensitive to dominance patterns.

Prior to multivariate analysis, the mosquito abundance matrix underwent Hellinger transformation (decostand, method = “Hellinger”) to reduce the influence of highly abundant species and to preserve Euclidean distance properties. PCA was then performed using the *rda* function in the vegan package [26] to explore patterns of community variation across habitats and seasons.

Statistical tests were performed to analyse differences in mosquito community composition among habitats and seasons, using permutational multivariate analysis of variance (PERMANOVA) based on *Bray–Curtis* dissimilarities. This was implemented using the *adonis2* function in the vegan package [27]. This approach quantified the proportion of variance in species composition explained by habitat type and seasonal variation. Finally, CCA was employed to investigate the relationship between the abundance of mosquito species and environmental and climatic variables. Based on the CCA outputs, a sensitivity score was calculated for each species to quantify its responsiveness to environmental variation, providing an integrative measure of species–environment relationships.

To determine the relative contributions of climatic, environmental, and anthropogenic variables, a variation partitioning analysis was performed using the *varpart* function in the *vegan* package. The variables were categorised as climatic (e.g. rainfall, air temperature, soil moisture, relative humidity), natural environmental (NDVI, elevation and proximity to water) or urbanisation (e.g. built-up density and proximity to built-up areas)-related predictors.

## Results

### Species diversity and domesticity index

A total of 6794 mosquitoes representing 24 species across 7 genera were collected (Table 1). The most abundant species were *Culex eumelanomyia* spp (*n* = 2447), *Cx. cinereus* (*n* = 1072), *Ae. albopictus* (*n* = 261), *An. paludis* (*n* = 23), and *An. funestus* (*n* = 20). Additional taxa, including *Coquillettidia* spp., *Eretmapodites* spp., and *Uranotaenia* spp., were recorded in lower abundance but contributed to overall species richness.

**Table 1:** Mosquito species diversity and domesticity index in the study area

	Mosquito species	Total (*n*)	Domesticity index (DI)
*Aedes*	*Aedes aegypti*	11	1
	*Aedes albopictus*	261	1
*Anopheles*	*Anopheles funestus*	20	1
	*Anopheles spp*	5	0.8
	*Anopheles gambiae s.l*	10	1
	*Anopheles paludis*	23	1
	*Anopheles coustani*	15	1
	*Anopheles nili*	27	1
	*Anopheles obscurus*	11	1
*Coquillettidia*	*Coquillettidia*	75	0.893
*Culex*	*Culex quinquefasciatus*	778	0.985
	*Culex eumelanomyia* spp	2447	0.997
	*Culex tigripes*	213	0.995
	*Culex cinereus*	1072	0.970
	*Culex annulioris*	26	0.770
	*Culex neavei*	347	0.986
	*Culex argenteopunctatus*	650	0.945
	*Culex macfiei*	365	1
	*Culex thalassius*	16	1
	*Culex duttoni*	40	1
*Mansonia*	*Mansonia uniformis*	42	0.976
	*Mansonia africana*	11	1
*Eretmapodites*	*Eretmapodites spp*	188	0.915
*Uranotaenia*	*Uranotaenia spp*	141	0.730

*DI* Domestic individuals/Total number of individuals; DI values close to 1 indicate a strong domestic association, while values close to 0 reflect a predominance in wild settings

The domesticity index (DI), which quantifies mosquito species’ affinity for domestic or peridomestic habitats, was generally high across sampled community. Several species displayed a DI equal to 1.0, indicating that all individuals were collected in human-altered environments. These species included *Ae. aegypti*, *Ae. albopictus*, *An. gambiae s.l.*, *An. funestus*, *An. paludis*, and several *Culex* species (*Cx. macfiei*, *Cx. thalassius*, and *Cx. duttoni*). Other *Culex* species also showed high DI values, typically exceeding 0.94. Lower indices were observed for *Uranotaenia* spp. (DI = 0.73), *Cx. annulioris* (DI = 0.77), *Coquillettidia* spp. (DI = 0.89), and *Eretmapodites* spp. (DI = 0.92). This indicates a comparatively weaker association with domestic habitats. These patterns emerged from a sampling design focused on urban and peri-urban environments, where traps were frequently deployed near human dwellings.

### Richness and diversity vary according to habitat and season

**Fig. 2:**
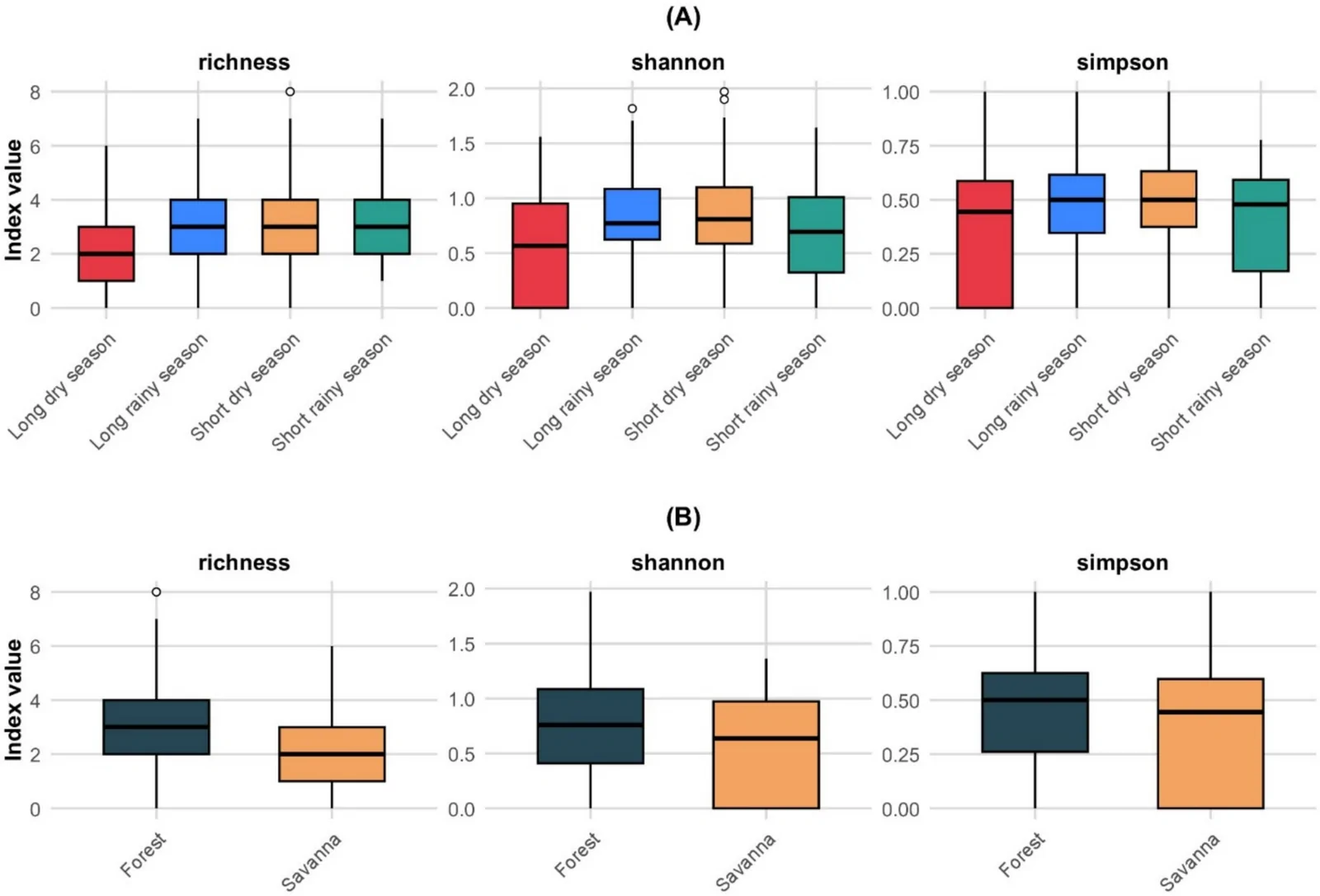
Diversity indices of mosquito communities by habitat and season. **A** Variation in species richness, Shannon, and Simpson diversity indices between forest and savanna habitats. **B** Comparison of the same diversity indices across the four climatic seasons (long rainy season, long dry season, short rainy season, short dry season). Boxplots show the distribution of index values, with the horizontal line representing the median. Seasonal categories correspond to the climatic cycle of southeastern Gabon: long rainy season (mid-February to mid-June), long dry season (mid-June to mid-September), short rainy season (mid-September to mid-November), and short dry season (mid-November to mid-February)

The richness and diversity of mosquito species differed across habitat types (Fig. 2A). Forest environments exhibited greater taxonomic complexity, as indicated by higher species richness and Shannon diversity (H’), than savannah environments. In contrast, the *Simpson* index revealed a more even distribution of species in the savannah, suggesting reduced dominance by a few taxa.

Distinct seasonal patterns were also observed (Fig. 2B). Species richness peaked during the long rainy season, reaching a maximum of 16 species and a median of 13 species. *Shannon* diversity indices were elevated during rainy periods, averaging approximately 2.3. In contrast, the *Simpson* index (D) indicated a more homogeneous species distribution, particularly during the short dry season (D approximately 0.84). The long rainy season exhibited the highest species richness (> 15 species), while the long dry season displayed the lowest diversity, with a maximum of eight species, a median of five, and an average *Shannon* index of approximately 1.2.

**Fig. 3:**
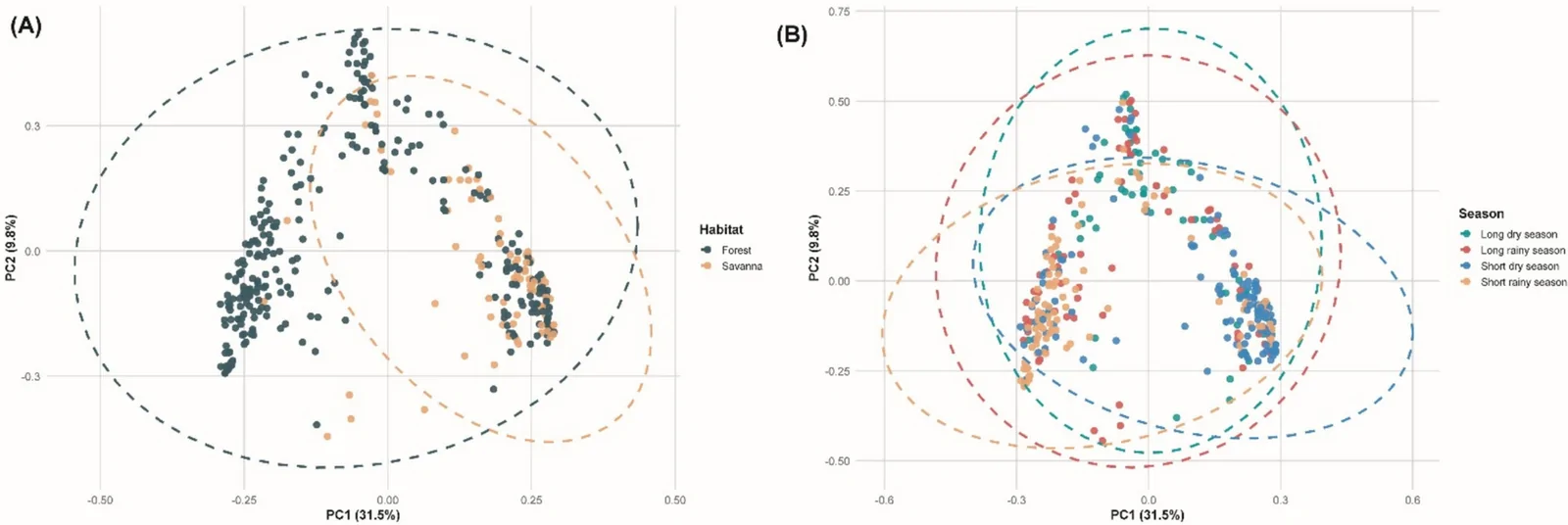
Mosquito community structure according to habitat and season. **A** PCA of mosquito community composition based on species abundance per sampling unit. Each dot represents a sampling unit, coloured according to habitat type. The ordination shows a tendency for forest and savannah communities to cluster separately. Ellipses represent the 95% confidence intervals of the group centroids. **B** PCA of mosquito community composition by season, using the same community matrix. Dots represent sampling units and are coloured according to season

Variations in community structure were corroborated by principal component analysis (Fig. 3A). PCA reveals habitat-driven separation along the first axis, explaining 31.5% of the variance, with forest samples clustering distinctly from savannah samples. This indicates a strong influence of habitat type on species composition. The second axis (PC2 = 9.8%) captured variability within forest communities. Seasonal ordination (Fig. 3B) shows partial overlap among seasonal groups. However, assemblages from the long rainy season and the short dry season tended to oppose each other along PC1.

### Ecological sensitivity of mosquito species

CCA was used to assess the relationship between the distribution of mosquito species and environmental and climatic factors (Table 2). Relative humidity, air temperature, and proximity to built-up areas were identified as the most influential factors in structuring species distributions. No significant spatial autocorrelation was detected for NDVI, temperature, or distance to buildings (*p* > 0.05). Although rainfall and relative humidity showed statistically significant Moran’s I values, their effect sizes were very low (*I* < 0.01), indicating weak spatial structuring. Overall, these results suggest that sampling sites can be considered largely independent of each other and that the 2 km buffer did not introduce strong spatial dependence.

**Table 2:** Canonical correspondence analysis loadings describing the contribution of environmental variables to mosquito species distributions (a negative sign indicates a negative contribution, and a positive sign indicates a positive contribution)

	Mosquito species	Relative humidity (%)	Air temperature (°C)	Distance to nearest building (m)	Distance from water (m)	Building density
*Aedes*	*Ae. aegypti*	−7.58	5.88	-0.45	3.23	0.11
	*Ae. albopictus*	−0.67	−0.07	−0.08	0.45	−0.16
*Anopheles*	*An. coustani*	0.42	3.45	−2.32	−0.38	−0.04
	*An. funestus*	−4.63	−2.64	2.10	−0.29	−0.93
	*An. gambiae* s.l	−1.58	−2.49	15.37	2.67	20.32
	*An. nili*	0.63	−0.26	−0.96	−0.98	−0.43
	*An. obscurus*	3.92	−4.06	3.00	−1.40	−0.56
	*An. paludis*	2.32	−2.19	−0.27	−0.94	0.44
	*Anopheles* sp.	−9.12	6.25	0.85	−0.28	−1.02
*Coquillettidia*	*Coquillettidia* sp.	0.48	−0.74	1.29	−0.57	−0.45
*Culex*	*Cx. annulioris*	3.37	−1.65	−0.52	−1.65	−1.94
	*Cx. argenteopunctatus*	0.00	0.18	−1.16	−0.21	0.75
	*Cx. cinereus*	−0.91	−1.01	−0.35	1.15	0.49
	*Cx. duttoni*	−4.24	6.82	−2.73	0.38	0.95
	*Cx. eumelanomyia* spp.	0.08	0.42	0.14	−0.82	−0.53
	*Cx. macfiei*	1.60	0.09	0.52	−0.44	−0.56
	*Cx. neavei*	0.88	0.73	1.90	−0.55	−0.61
	*Cx. quinquefasciatus*	−0.33	0.54	−0.73	1.72	1.00
	*Cx. thalassius*	3.11	−3.27	−1.97	−1.58	0.86
	*Cx. tigripes*	1.79	−1.44	−0.26	1.35	0.15
*Eretmapodites*	*Eretmapodites* sp.	1.56	−2.13	0.61	−1.13	−0.50
*Mansonia*	*Mansonia africana*	0.61	−0.92	1.84	−0.83	−1.17
	*Mansonia uniformis*	−1.57	0.19	0.34	−0.75	−0.40
*Uranotaenia*	*Uranotaenia* sp.	−1.31	−0.99	2.22	−0.78	−0.60

Mosquito species, such as *Anopheles gambiae s.l.*, *An. Funestus,* and *An. obscurus* exhibited strong positive associations with air temperature and built-up density, as well as negative associations with relative humidity. In contrast, most *Culex* and *Mansonia* species exhibited weak correlations with the environmental variables, as indicated by low loadings on the main CCA axes. *Aedes aegypti* was positively associated with temperature and negatively with humidity, whereas *Ae. albopictus* displayed more balanced and moderate associations across environmental gradients.

**Fig. 4:**
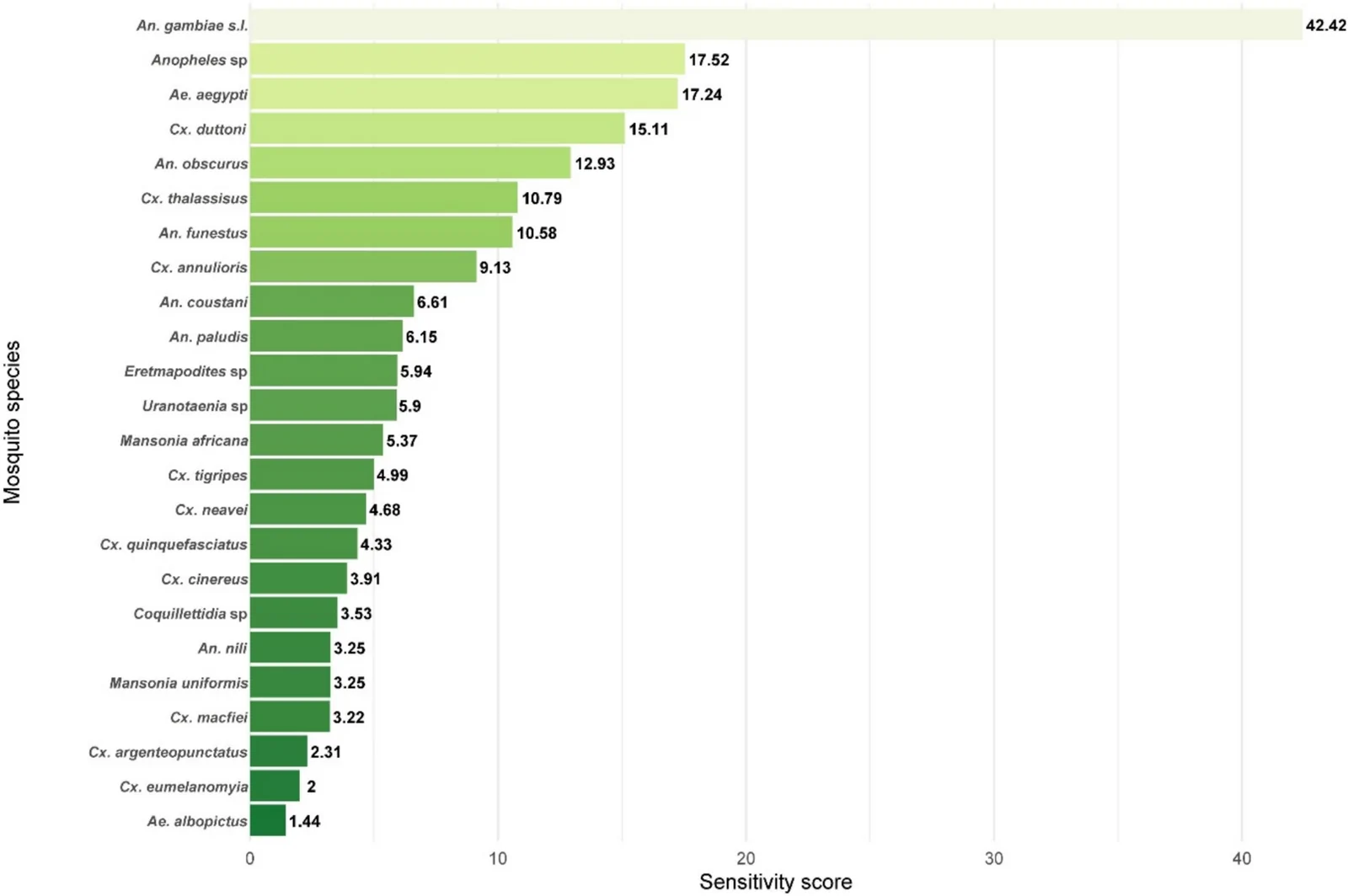
Environmental sensitivity of mosquito species (sum of absolute canonical coefficients)

Ecological sensitivity scores derived from the CCA loadings (Fig. 4) varied substantially among species. The highest scores were recorded for *An. gambiae s.l.* (42.4), *An.* sp. (17.5), *Ae. aegypti* (17.2), *Cx. duttoni* (15.1), and *An. obscurus* (12.9). Intermediate scores were obtained for *An. funestus*, *Cx. Cinereus,* and *Mansonia uniformis.* The lowest sensitivity scores, below 2.5, were found in *Ae. albopictus*, *Cx. Eumelanomyia,* and *Cx. argenteopunctatus*.

### Relative contributions of climatic, environmental, and urban drivers

**Fig. 5:**
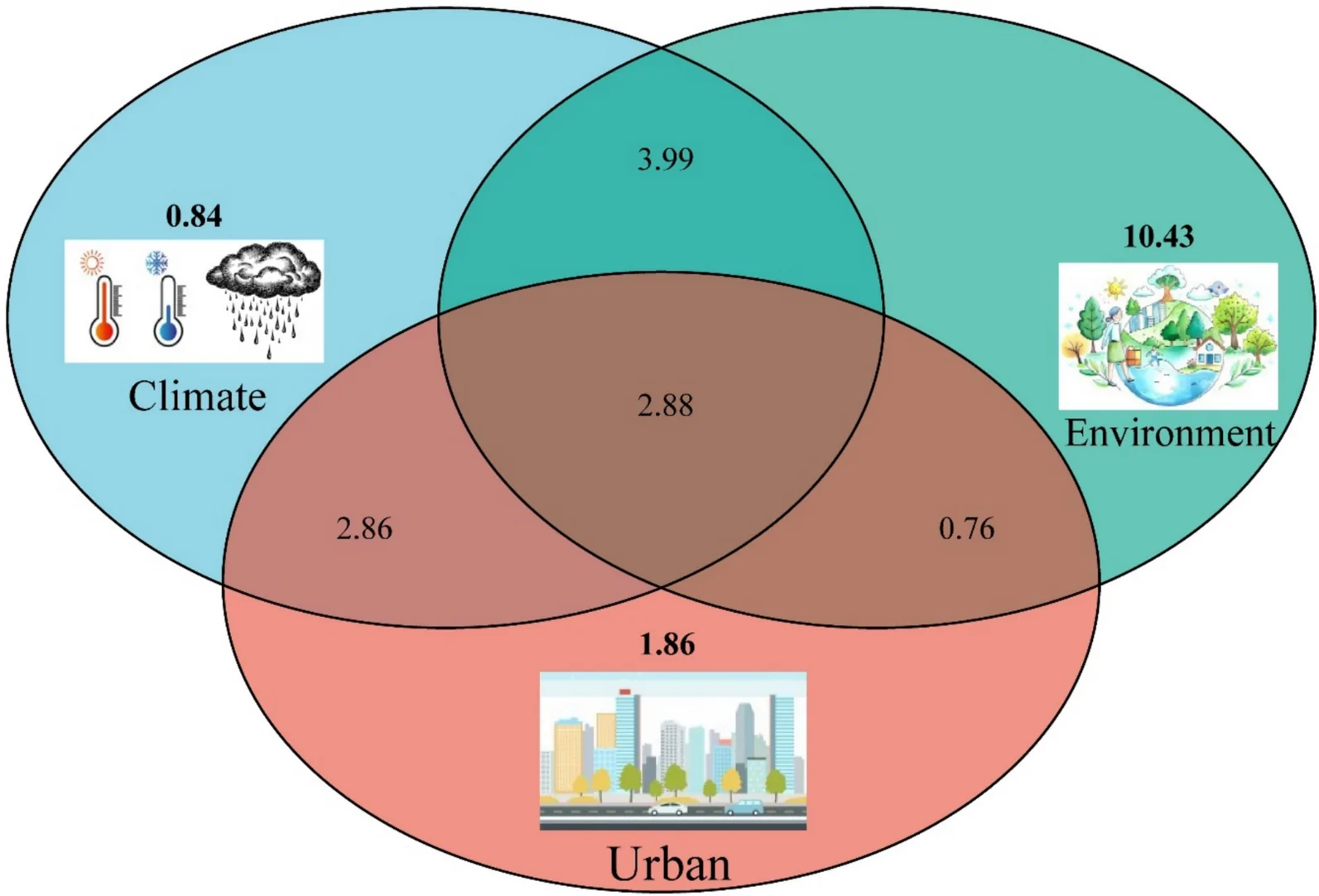
Variation partitioning of mosquito community composition across climatic, environmental, and urban predictor groups

Variation partitioning analysis (Fig. 5) showed that natural environmental variables accounted for the largest proportion of unique variance in the mosquito community (10.43%). Climatic predictors contributed 0.84%, while urbanisation-related factors explained 1.86%. The substantial shared variance among the predictor groups highlights the interactive structuring of mosquito assemblage within the heterogeneous intra-urban landscape. Overall, the combined model explained 23.62% of the total community variance, leaving 76.38% unexplained.

## Discussion

### Global patterns of mosquito community structure in an intra-urban context

Effective control of vector-borne diseases requires an understanding of how mosquito populations change over time and space, since changes in species, numbers, and habitat affect the risk of disease spread [28]. This study provides a detailed ecological assessment of mosquito communities and their environmental factors in and around the CIRMF area in Franceville. This secondary city features a mix of forest and savannah patches within the urban area. The study area, fully inside Franceville’s urban boundaries, encompasses homes, institutional buildings, and residual forest patches near the CIRMF, including its Primatology Centre. This mix forms a human environment interface that is important for disease studies. Captive non-human primates and surrounding forest habitats link humans, wildlife, and mosquitoes, with documented cases of disease such as human *Plasmodium* spp. passing to chimpanzees [29].

The findings reveal a diverse mosquito assemblage numerically dominated by the genus *Culex*, while also including medically important *Aedes* and *Anopheles* vectors. This is consistent with previous studies conducted in mixed landscapes [30–32]. The presence of species with diverse ecological requirements and adaptability underscores the complexity of mosquito communities in this heterogeneous urban environment and highlights the importance of surveillance based on ecological knowledge.

### Species diversity and community composition

We collected 6794 mosquitoes belonging to seven genera, indicating substantial mosquito diversity within the urban landscape of Franceville. Species composition varied markedly across habitats and seasons. Forest sites consistently harboured higher species richness and diversity than savannah sites, with diversity peaking during the long rainy season. Similar patterns have been reported in other tropical urban environments, where landscape heterogeneity and seasonal rainfall strongly influence mosquito community structure [33–35]. These patterns were further supported by the PCA results, which revealed a clear, habitat-driven separation of mosquito assemblages. The first principal component alone accounted for 31.5% of the total variance, clearly separating forest and savannah communities. This indicates that habitat type is a significant factor in the ecological structuring of the study area. Furthermore, the second axis (explaining 9.8% of the variance) revealed significant variability within forest habitats, suggesting that forest patches vary in their microenvironmental characteristics and ecological suitability for different mosquito species.

Despite this diversity, communities were numerically dominated by *Culex* species, particularly *Cx. Eumelanomyia* spp, and *Cx. cinereus*. Such dominance by *Culex* species has been documented in several equatorial African urban settings and likely reflects the broad ecological plasticity of this genus and its capacity to exploit human-modified environments [30–32]. Other *Culex* species recorded (*Cx. macfiei*, *Cx. Thalassius,* and *Cx. duttoni*) further illustrate the adaptive flexibility of this genus.

We still lack detailed information on the host preferences of many species. Some seem to prefer birds, whereas others feed opportunistically on both birds and mammals [36]. *Culex duttoni* appear near humans, but its host preferences are not well known [30, 37]. Data on feeding ecology are also scarce for dominant species such as *Cx. Eumelanomyia* spp and *Cx. cinereus* [38]. This gap makes it hard to fully understand their role in disease spread in Central African cities.

Importantly, several mosquito species recorded in the present survey, including *Culex duttoni*, *Cx macfiei*, *Cx. Thalassius*, and *Cx. Argenteopunctatus*, have rarely been reported in previous entomological surveys conducted in Gabon [39]. Their detection likely reflects the broader ecological coverage and taxonomic resolution achieved in the present study rather than recent introductions. These findings enhance our understanding of the mosquito fauna in Franceville and highlight the importance of local surveys in rapidly changing urban areas.

### Habitat- and season-driven structuring

There were clear changes in mosquito communities across space and time. Forest habitats had a greater number of species and higher diversity scores, reflecting a more complex environment and greater availability of niches. These forests provide shade, higher humidity, and diverse larval habitats, helping species coexist. Nevertheless, some species were dominant, likely due to resource sharing and interactions [20]. Savannah habitats had fewer species, but more balanced communities as indicated by Simpson’s index. The greater temperature and humidity fluctuations experienced in these open environments potentially limit diversity but reduce competition among dominant species.

Seasonal effects were equally pronounced. Species peaked during the long rainy season, confirming the central role of rainfall in creating and maintaining larval habitats [40, 41]. Diversity declined during the long dry season due to harsh climate conditions. These patterns indicate strong seasonal shift in mosquito assemblages, which could affect how diseases spread [42–44]. Seasonal ordination analyses also revealed a partial segregation between communities sampled during the long rainy season and those collected during the short dry season. This pattern suggests that seasonal variation influences not only mosquito abundance and diversity but also species composition, reflecting seasonal turnover in community structure.

### Environmental heterogeneity and species-specific ecological sensitivity

Our analysis revealed contrasting ecological responses among mosquito genera. *Anopheles* species exhibited the highest ecological sensitivity, reflecting a stronger dependence on specific environmental conditions. In particular, *Anopheles gambiae s.l.* showed clear associations with humidity-related variables and proximity to aquatic habitats, consistent with its well-established ecological requirements [45]. Although *Anopheles* species were detected at relatively low abundance in our survey, this pattern cannot be attributed solely to the availability of larval habitats. Multiple ecological factors may contribute to their distribution and abundance within the urban landscape, including host availability, dispersal capacity, habitat fragmentation, microclimatic conditions, and local environmental suitability.

Recent studies have nevertheless documented increasing ecological plasticity in urban populations of *An. gambiae s.l.* and *An. funestus*, including adaptation to polluted, artificial, or otherwise unstable breeding sites [46, 47]. However, the extent of this plasticity is likely to vary among locations and may not fully offset local ecological constraints operating within the heterogeneous intra-urban landscape of Franceville. Consequently, both environmental filtering and adaptive capacity should be considered when interpreting the distribution of urban malaria vectors and designing vector control strategies [45, 48, 49].

In contrast, most *Culex* and *Aedes* species exhibited lower sensitivity scores, indicating broader ecological tolerance. The low sensitivity of *Culex quinquefasciatus* and *Cx. cinereus* illustrates their ability to persist across heterogeneous urban habitats [30, 50]. Interestingly, *Cx. duttoni* exhibited a relatively high sensitivity score (15.1), contrasting with the generally low sensitivity observed among most *Culex* species. Although little is known about its ecology in Central Africa, this result suggests a stronger dependence on specific environmental conditions and highlights the need for further ecological investigations of this poorly documented species. This ecological ubiquity may facilitate the circulation of *Culex*-borne pathogens across diverse urban microenvironments [51–53].

The findings of this study showed that natural environmental factors account for most of the differences in mosquito communities, rather than climate and urbanisation alone. Variation partitioning analysis provided further evidence that natural environmental variables were the primary drivers of mosquito community structure, accounting for 10.43% of the uniquely explained variance. In contrast, climatic variables and urbanisation-related predictors explained only 0.84% and 1.86% of the variance, respectively. These results indicate that local habitat characteristics exert stronger ecological filtering than broader climatic conditions or urban infrastructure within the study area. This suggests that small-scale ecological heterogeneity within cities shapes mosquito groups more than broader climate or built density indicators. Studies show that land cover, plants, and water features are better predictors of mosquito presence than general climate indicators [54, 55]. These local habitat characteristics, including vegetation patches and varied larval sites, create niches that support distinct mosquito assemblages, more effectively than broad climatic or built environment measures do. This is also suggested for urban green spaces [56]. Importantly, the shared fractions among predictor groups further emphasise the interactive nature of ecological and anthropogenic drivers. Recent work in urban mosquito ecology highlights that climatic, land use, and landscape features jointly influence vector assemblages jointly, rather than independently [57–59]. The substantial proportion of shared variance observed among predictor groups further suggests that environmental conditions, climatic variability, and anthropogenic modifications operate synergistically rather than independently. Such interactions likely contribute to the complex spatial organisation of mosquito communities observed within the intra-urban forest-savannah mosaic of Franceville.

### Epidemiological implications in a heterogeneous intra-urban landscape

The detection of *Aedes* species, particularly *Ae. albopictus*, is of epidemiological significance given its efficiency in transmitting arboviruses and its rapid expansion across Central Africa [60–63]. Its moderate sensitivity score indicates ecological flexibility, allowing it to persist in both urban and semi-natural habitats. This ecological flexibility may partly explain the successful expansion of *Ae. albopictus* throughout Central Africa and reinforces concerns regarding its capacity to invade newly urbanised landscapes while maintaining populations in heterogeneous environments.

Although *Anopheles* species were detected at low abundance, the occurrence of confirmed malaria vector, including *An. gambiae s.l.* and *An. funestus*, indicates a persistent risk of malaria transmission within Franceville. The domesticity index (DI) was generally high across species, indicating frequent occurrence in human-associated environments. However, the DI reflects habitat association rather than host preference and should therefore not be interpreted as direct evidence of anthropophilic feeding behaviour. Determining host preferences would require blood meal analysis, which was beyond the scope of this study. Sampling sites included forest patches adjacent to residential areas, reflecting the fragmented and interspersed nature of natural habitats within the city. Consequently, high domesticity values likely characterise human-associated and ecotonal assemblages rather than strictly domestic mosquito populations. Furthermore, the DI reflects habitat association rather than host-feeding preferences and should therefore not be interpreted as direct evidence of anthropophilic behaviour. Determining host preferences would require blood meal analyses, which were beyond the scope of the present study.

The presence of remnant forest patches within the urban fabric, particularly around the Primatology Centre, may enhance ecological connectivity among mosquito populations inhabiting human-associated and wildlife-associated habitats. Such landscape configurations may promote spatial overlap among vectors, humans, and wildlife hosts. However, the extent to which individual mosquito species contribute to pathogen circulation across this interface remains to be investigated. Integrating ecological heterogeneity into surveillance frameworks is therefore essential for improving our understanding and management of vector-borne disease risks in rapidly transforming secondary cities.

### Study limitations and perspectives

Several limitations require consideration, as do promising directions for future research. For example, CDC light traps preferentially capture nocturnal, host-seeking mosquitoes, which could lead to an underrepresentation of diurnal or container-breeding taxa [64]. This sampling bias could result in inaccurate estimates of overall mosquito diversity and abundance. Furthermore, trap performance may vary across habitats and climatic conditions, affecting the comparability of relative abundance estimates between sites.

The configuration of the sampling sites, consisting of forest patches within an urban matrix, reflects the ecology of Franceville but may have inflated domesticity indices by capturing mosquitoes from both sylvatic and domestic environments. Therefore, these values should be interpreted as indicative of ecotonal intra-urban communities. Nonetheless, several research gaps persist. For example, although spatial autocorrelation indicates weak site dependence, proximity between sites may constrain inferences regarding very fine-scale spatial variation. Additionally, the low abundance of certain taxa, particularly *Anopheles*, may exaggerate apparent sensitivity to environmental variations. Further research is necessary to assess how these limitations influence the generalizability of the findings to other urban contexts and taxonomic groups.

It is important to note that this study focused on community structure and environmental drivers but did not evaluate pathogen infection rates. This limitation offers a significant opportunity for future research to integrate entomological, parasitological, and virological surveillance alongside complementary larval and diurnal sampling methods. Such a multidisciplinary approach could provide novel insights into transmission dynamics within heterogeneous intra-urban environments such as Franceville, thereby informing the development of more effective and ecologically based vector control strategies.

## Conclusions

This study reveals a highly diverse community of mosquitoes, dominated primarily by *Culex* species, alongside *Aedes albopictus* and several medically significant *Anopheles* species. This diversity reflects a complex ecological mosaic, with each genus occupying a distinct adaptive niche. *Anopheles* mosquitoes are highly sensitive to their environment, which is closely linked to the specific climatic and habitat conditions that facilitate malaria transmission. Conversely, *Culex* species demonstrate notable ecological plasticity, as evidenced by their numerical dominance and consistent presence across the various habitats surveyed, including anthropised forest patches and savannah environments. *Aedes albopictus* combines adaptability with strong invasive potential, posing an increasing threat to the transmission of arboviruses. These patterns illustrate the coexistence of sensitivity and resilience within the same landscape, suggesting that the risk of vector-borne diseases will likely increase and persist amid environmental change. Consequently, it is essential to integrate ecological and climatic variability into surveillance frameworks. A holistic, ecosystem-based approach to vector management is necessary to anticipate and effectively control emerging risks effectively in rapidly developing secondary urban areas in tropical regions such as Franceville.

## Data Availability

Data supporting the main conclusions of this study are included in the manuscript.

## Abbreviations

*Ae*.: *Aedes*
*An.*: *Anopheles*
CCA: Canonical correspondence analysis
CDC: Centers for disease control
CIRMF: Centre Interdisciplinaire de Recherches Médicales de Franceville
*Cx*.: *Culex*
D: *Simpson* Index
DI: Domesticity index
H’: Shannon diversity
NDVI: Normalized difference vegetation Index
PCA: Principal component analysis
*s.l*: *sensus lato*
URES: Unité de Recherche en Ecologie de la Santé du CIRMF
VarPar: Variation partitioning
